# Sliding Wear Behavior of UNS R56400 Titanium Alloy Samples Thermally Oxidized by Laser

**DOI:** 10.3390/ma10070830

**Published:** 2017-07-19

**Authors:** Juan Manuel Vazquez Martinez, Francisco J. Botana Pedemonte, Marta Botana Galvin, Jorge Salguero Gomez, Mariano Marcos Barcena

**Affiliations:** 1 Department of Mechanical Engineering & Industrial Design, Faculty of Engineering, University of Cadiz, Av. Universidad de Cadiz 10, E-11519 Puerto Real-Cadiz, Spain; jorge.salguero@uca.es (J.S.G.); mariano.marcos@uca.es (M.M.B.); 2 Department of Materials Science and Metallurgic Engineering and Inorganic Chemistry, Faculty of Engineering, University of Cadiz. Av. Universidad de Cadiz 10, E-11519 Puerto Real-Cadiz, Spain; javier.botana@uca.es (F.J.B.P.); marta.botana@uca.es (M.B.G.)

**Keywords:** UNS R56400, laser oxidation, sliding wear, wear mechanism, tribology, hardness, roughness, pin on disc

## Abstract

Wear of elements subjected to friction and sliding is among the main causes of low tribological performance and short lifetime of strategic materials such as titanium alloys. These types of alloys are widely used in different areas such as aerospace and the biomechanics industry. In this sense, surface modification treatments allow for the overcoming of limitations and improvement of features and properties. In the case of titanium alloys, improvements in the main weaknesses of these materials can be obtained. Laser texturing of UNS R56400 (Ti6Al4V) alloy, according to Unified Numbering System designation, surface layers in a non-protective atmosphere produces an increase of the oxides, especially of titanium dioxide (TiO_2_) species. The presence of oxides in the alloy results in color tonality variations as well as hardness increases. In addition, specific roughness topographies may be produced by the track of laser beam irradiation. In this research, thermochemical oxidation of UNS R56400 alloy has been developed through laser texturing, using scan speed of the beam (Vs) as the process control variable, and its influence on the sliding wear behavior was analyzed. For this purpose, using pin on disc tribological tests, wear was evaluated from the friction coefficient, and wear mechanisms involved in the process were analyzed. Combined studies of wear mechanisms and the friction coefficient verified that by means of specific surface treatments, an increase in the wear resistance of this type of alloys is generated. The most advantageous results for the improvement of tribological behavior have been detected in textured surfaces using a Vs of 150 mm/s, resulting in a decrease in the friction coefficient values by approximately 20%.

## 1. Introduction

UNS R56400 (Ti6Al4V) titanium alloy is one of the strategic materials in the aerospace industry, mainly due to the ratio between physicochemical properties and weight [1,2,3,4]. In addition, excellent biocompatibility with living organisms is presented, making this alloy one of the most commonly used in the manufacture of prostheses and biomechanical components [1,2]. However, under tribological wear conditions, driven primarily by a low plastic deformation ratio, titanium alloys do not show good work performance, presenting fluctuations in friction coefficient (μ) values and unwanted wear mechanisms [1].

Surface modification procedures, especially thermo-chemical treatments, allow variations in the microstructure and composition of the initial surface of the alloy, developing layers with specific properties and characteristics. According to [5,6,7,8,9,10,11,12,13,14], these changes can improve the surface integrity of the alloy, increasing the functional features in order to adapt the material to the requirements of the current industry.

In particular, preliminary studies have confirmed that surface oxidation treatment of Ti6Al4V alloy by laser treatments, enables the generation of samples with modified surface features, giving rise to, among others, variations in aesthetic appearance (color tonality), surface finish (roughness), and hardness or tribological behavior [15,16,17,18]. Also, the minimally invasive conditions of some of the surface treatments, such as lasers, allow for the maintenance of good control over the thickness of the treated layer, keeping intact the initial properties of the remaining material [15,16,17,18,19]. However, cooling processes involved in thermal treatments may be the cause of instabilities in the microstructure of titanium alloys. This fact is especially due to volume variations between the oxides and the material of the initial alloy. In this aspect, according to Pilling-Bedworth ratio [20,21] for higher oxide volume, the process produces mechanical stress, making the outer layer fragile and favoring the possibility of fractures over the surface [21,22,23,24,25].

Surface treatments are important for the improvement of tribological behavior of mechanically contacted elements with relative movement; in these cases, it is hard to ensure efficiency in service over a prolonged lifetime. The use of lubricants and coolants reduce the friction effects, facilitating the sliding displacement. However, mainly due to current environmental restrictions, reduction of the fluids used is among the main objectives of most fields of the industry. In this regard, the study of surface treatments aimed at reducing the effects of wear shows great importance in improving the friction wear behavior of materials [5,26]. Due to the impossibility of maintaining control over a large number of variables in the cutting and forming processes, the study of behavior under wear and friction conditions is usually carried out by tribological tests designed to reproduce the conditions of the material in controlled sliding wear situations. In this sense, these types of tests allow for the maintenance of an accurate registration process of the main variables involved in the process [26,27,28,29].

The main objective of this work is to investigate the influence of surface treatments by laser texturing in the improvement of the wear behavior of the Ti6Al4V alloy, focusing interest on the effects produced by the present wear mechanisms. In order to carry out the changes, a layer of modified material has been developed by laser texturizing techniques, varying the properties and initial features directly related to friction wear, such as hardness and surface finish, and its influence has been evaluated on the contact conditions of the tribological pair and wear material rate. Located wear mechanisms in the sliding process and their relationship with the friction coefficient have been identified as an indicative control variable for the reduction of wear effects of the modified alloy.

## 2. Materials and Methods 

UNS R56400 Ti alloy sheets (60 × 60 × 5 mm^3^) have been used to carry out this research. Using Optical Emission Spectrometry (OES), the composition of the starting alloy has been evaluated, obtaining the values indicated in Table 1. By a mechanical polishing process, the surface of the samples has been adapted to roughness values of Ra < 0.05 μm/Rt < 0.20 μm/Rpk < 0.075 μm.

### 2.1. Laser Texturing Process

**Figure 1:**
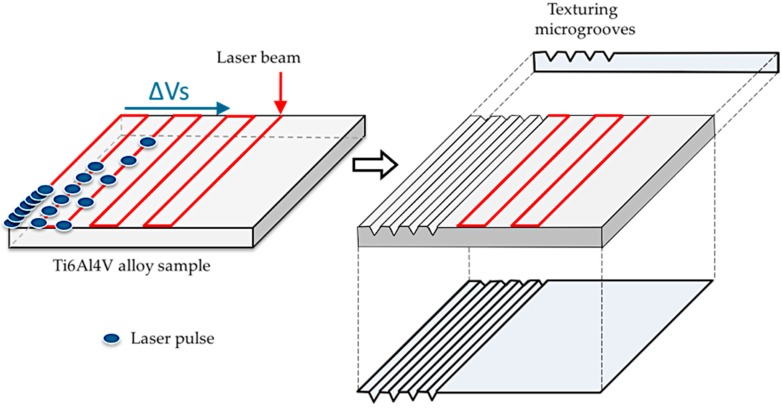
Layout and effects of laser texturing over Ti6Al4V samples.

Texturing procedure has been carried out over the surface of the Ti6Al4V samples using an Ytterbium fiber infrared pulsed laser of 1070 ± 5 nm wavelength, model Rofin EasyMark F20. Bidirectional ways layout without overlapping of laser irradiation has been described throughout the study samples, resulting in parallel textured traces with approximately 0.1 mm separation distance between laser ways. Texturization treatments have been carried out using a 60 μm spot diameter in an open-air atmosphere, favoring the oxidation of the modified layer. By means of variations of the scan speed of the laser beam, treatments with different intensity have been developed, increasing the aggressiveness as a function to the number of pulses and the time that the laser beam remains over a section of the sample, Figure 1. In this way, lower scanning speeds result in a greater supply of energy on the surface.

Two types of textured samples have been developed in this experimental methodology. A first group of experimental probes was designed for the characterization of properties and features of the modified layer, and a second group was designed for the study of the tribological behavior thereof. Texturing conditions of both specimens in terms of atmosphere under which the process took place and the equipment used were the same. The specimens varied in the laser treatment parameters of pulse rate, F, and scan speed of the beam, Vs, Table 2. All treatments have been carried out at environmental temperature, producing relatively fast cooling stages of the textured zone.

### 2.2. Textured Layer Characterization

The characterization of the material modified layer focused on surface finish and hardness parameters, as well as tonality color variations observed over the irradiated layer. For the evaluation of the surface finish resulting from the textured processes, a Mahr Perthometer Concept PGK120 measurement station was used to measure the roughness of at least ten profiles in the perpendicular direction to the laser beam traces, uniformly distributed on the entire treated surface.

For the study of surface finish evolution, reduced peak height parameter (Rpk) extracted from the material ratio or Abbott-Firestone curve [30] was selected. The Rpk parameter defines the average height of the peaks above the line of the roughness core profile. This parameter represents the fraction of material with a tendency to be eliminated in the first instants under the effect of sliding contact and, therefore, shows great information about the tribological behavior of the textured surface.

In addition, with the aim to observe in greater depth the resulting surface in terms of both the new microgeometry and the possible presence of oxygen generated by the laser beam under air atmosphere, a surface inspection was carried out using Scanning Electron Microscopy (SEM) and Energy Dispersive Spectroscopy (EDS) combined with the study of cross sections of the samples using Optical Microscopy (OM), this provided an accurate view of the shape and depth of textured grooves.

Due to the generated topography during the texturing process, in order to obtain more accurate reducing deviations from indentations over laser tracks, hardness measurements of the modified layer were carried out on the cross sections of the samples. Using a Struers Duramin-20 durometer (Westlake, OH, USA), Vickers hardness (HV) of the samples was determined by fixing a 245.2 × 10^−3^ N load (equivalent to 0.025 HV) and a time of 10 s as the test conditions. 

To ensure measurement goodness in the obtained values of hardness, after each of the measurement series, an additional measurement was made on an untreated sample, verifying that the results are close to the nominal hardness values of the Ti6Al4V alloy, and discarding measurement series with greater deviation than 5% of the nominal value of the material. Because the laser was used in an open-air atmosphere, the appearance of oxides (Oxygen) in the modified material layer was evaluated using SEM/EDS. Although it is not the main objective of this work, the importance of small increases in the concentrations of oxygen in titanium alloys can have a great impact on their mechanical properties, particularly in hardness [31]

### 2.3. Pin on Disc Tribological Tests

Once the characterization of the textured alloying layer was realized, Pin on Disc (PoD) tests were carried out, setting a 20 N load and 1000 m of sliding distance as the test conditions. Tungsten carbide (WC-Co) spheres with 3 mm diameter were used as the pin. This type of pin has been selected because the tribological pair formed by the two materials (WC-Co + Ti6Al4V) is among the most common in the cutting processes of the current industry. Subsequent to the tests, a SEM/EDS study of the elements involved in the sliding contact was developed, analyzing the effects of friction and sliding and identifying the main wear mechanisms that take place in these types of processes. 

Repeatability deviations of the measurements in all parameters evaluated in the present paper are not significant in relation to the obtained values, in this way, these deviations have not been represented explicitly in the graphs.

## 3. Results and Discussion

Modification procedures through laser texturing resulted in the variation of the initial surface integrity of the alloy. Moreover, due to the irradiation of the material in a non-protective atmosphere, the introduction of oxides in the titanium alloy (Ti*_x_*O*_y_*) was favored, showing higher concentrations in the form of titanium dioxide (TiO_2_) species. The oxidation of the textured surface layer, in combination with microstructural changes mainly produced by cooling processes that take place in the developed thermo-chemical treatments, also caused modifications of the nominal values of hardness of the alloy.

Roughness and hardness of the surface finish are shown as parameters of great influence on the tribological behavior of the modified layer, being of special importance to the wear mechanisms present during the sliding stage of the contact elements.

**Figure 2:**
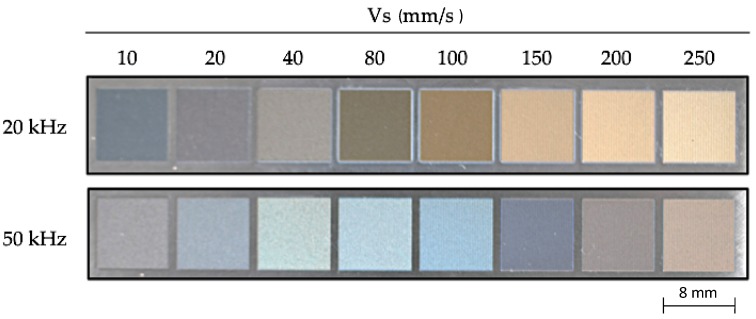
Surface tonality variations as a function of laser textured parameters.

Initially, the composition of the starting material was analyzed, verifying that the obtained value of components was in accordance with the limits established by the different standards for the Ti6Al4V alloy. After laser textured processing of the samples, very remarkable changes of tonality were observed on the surface, mainly due to the oxidation of the treated layer. Starting from the tonality variation, a notable influence on the nature of the modified alloy layer can be seen, which is especially caused by the laser treatment parameters pulse rate (F (kHz)) and scan speed of the beam (Vs (mm/s)), Figure 2. In accordance to [19,32], it has been found that for more aggressive texturizing treatments (lower pulse rate and Vs) in which higher thicknesses of modified layers are produced, there is a tendency towards tonalities of bluish and grayish color. On the other hand, when the intensity of the textured treatment is reduced by increasing Vs and/or reducing the pulse rate, color tonalities that can range from brown to golden textures are generated for those combinations of parameters with lower thickness of the modified layer. 

**Figure 3:**
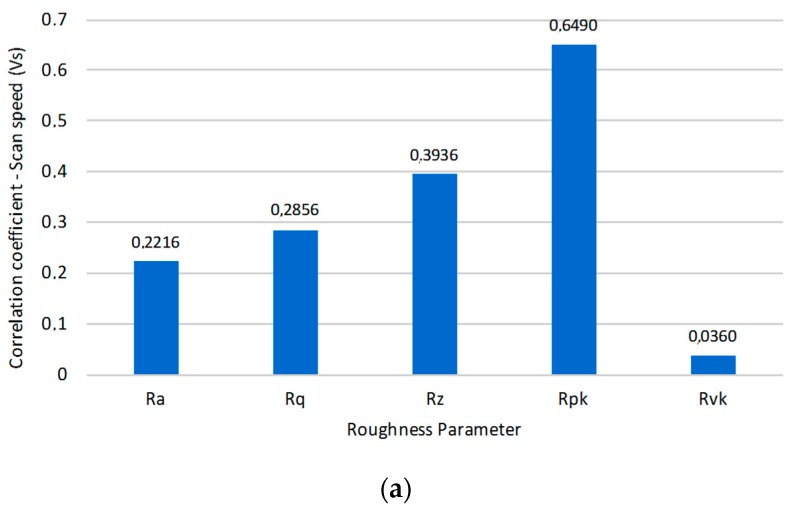

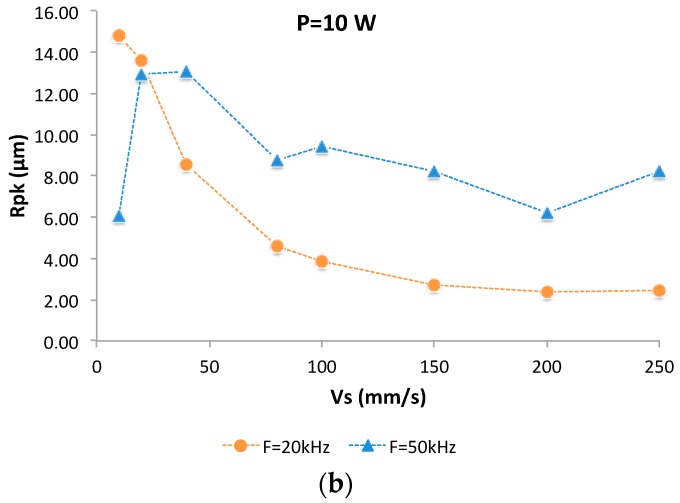
(**a**) Correlation between roughness parameters [33] and scan speed of the laser beam; (**b**) Roughness (Rpk) as a function of scan speed (Vs).

Also, surface finish is affected by texturing treatment. The laser beam track results in the formation of parallel longitudinal microgrooves on the surface of the alloy sample. In this way, variations of the texture parameters result in variations on the obtained roughness values. Although several parameters of roughness have been analyzed, Rpk parameter, due to its direct relation with the tribological characteristics of the surface, has been analyzed in depth, showing a direct relation to the scan speed of the beam with regard to other surface finish parameters. This fact has been confirmed by correlation analysis between Vs and roughness. In this sense, a remarkable decrease of the values of Rpk as a function of Vs may be observed. In this case, less aggressive treatments are produced by an increase of the scan speed, softening the generated topography and resulting in lower height asperities; Figure 3a,b.

**Figure 4:**
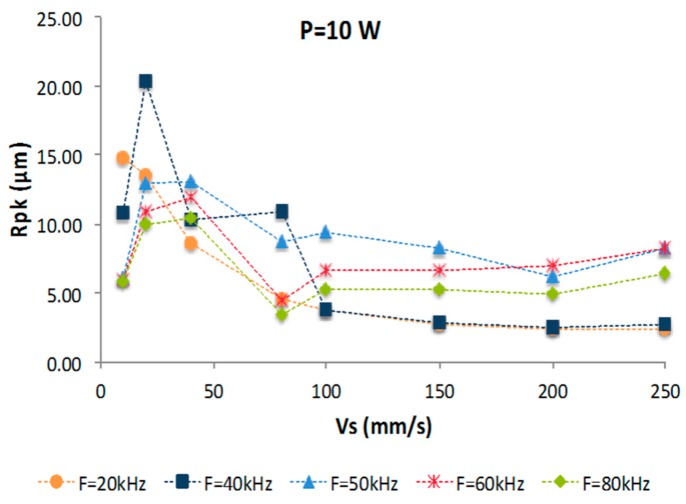
Roughness (Rpk) evolution as a function of scan speed (Vs) at different pulse rates (F).

Although pulse rate (F) maintains an important correlation with the roughness of the textured layer, from the point of view of the Rpk parameter, selected in this contribution for its relevance in the study of tribological behavior, a higher correlation can be observed in Vs with respect to the pulse rate. In this way, a correlation value of 0.1499 was obtained for Rpk as a function of F, and a correlation value of 0.6490 was obtained for Rpk as a function of Vs. In addition, roughness behavior (in terms of Rpk) was analyzed at pulse rates of 20, 40, 50, 60, and 80 kHz, maintaining significantly similar trends in all cases, as can be seen in Figure 4.

The Rpk parameter is defined by the average height of the peaks above the line of the roughness core profile, as such, the influence regarding the scanning speed is due to the development of larger asperities from lower speed beams at higher energy inputs of the same section of the alloy. For the case of average roughness parameters such as Ra or Rq, a clear dependence on Vs was not detected. This is mostly due to the periodic arrangement of the generated grooves by the laser process and its resulting geometry, which may have irregular sections from non-uniform cooling processes.

**Figure 5:**
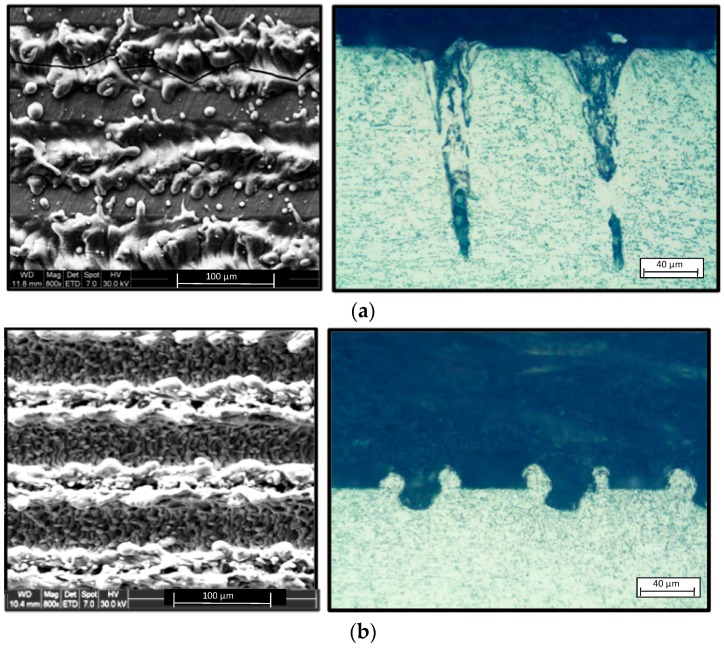
Surface microgeometry evolution (**a**) F = 50 kHz-Vs = 10 mm/s; (**b**) F = 50 kHz-Vs = 250 mm/s.

By SEM microscopy, the generation of a specific topography in connection with texturization parameters can also be observed on the treated surface. On the other hand, in order to study the depth and shape of the grooves, cross section inspection of the irradiated samples was carried out, resulting in important variations strongly influenced by scan speed of the beam, Figure 5a,b.

**Figure 6:**
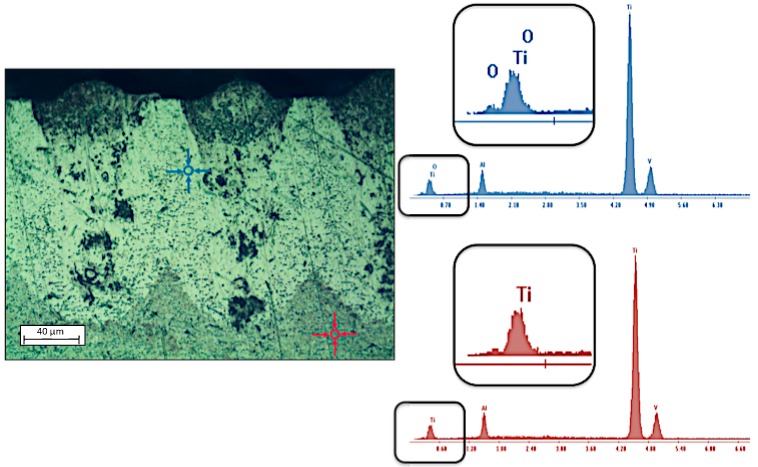
Oxygen presence of areas near textured grooves.

Through the study of cross sections of the textured samples, the existence of an area close to the laser generated grooves has been observed that shows different features to those of the substrate alloy. After a chemical etching process using Kroll’s method and the analysis of affected zone by dispersive energy spectroscopy (EDS), the presence of oxides (Oxygen) was detected, Figure 6. Thermochemical oxidation from the absorbed radiation in an air atmosphere also produces variations in properties such as hardness of the material.

**Figure 7:**
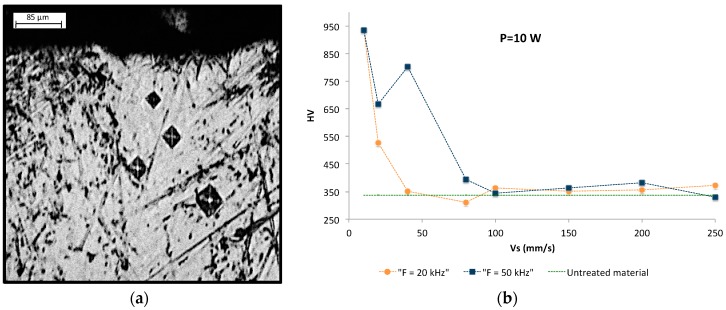
(**a**) Hardness gradient on textured groove; (**b**) Hardness (HV) as a function of scan speed (Vs).

Hardness of the material shows an increasing trend as a function to the time that the laser beam remains on the same section of material, i.e., for lower scanning speeds. This fact indicates that for higher thicknesses of the irradiated layer when affected by oxidation, a hardening phenomenon occurs which can triple the nominal hardness value of Ti6Al4V alloy. Increases in hardness values was also observed towards the area of direct incidence of the laser beam in the textured track. This effect can be verified through hardness gradients made along the section of the microgrooves, where smaller indentations were observed maintaining the same hardness test conditions, Figure 7.

The modification of surface properties and characteristics through laser texturization processes has direct effects on the tribological behavior of the alloy, showing special influence on the friction coefficient and wear mechanisms that are developed during the friction and sliding of the materials in contact.

It can be seen that the untreated sample shows an increasing trend over time in the coefficient of friction caused by the lack of protection of the oxide film. However, these layers are not particularly beneficial to the lower Vs as it has been detected. 

**Figure 8:**
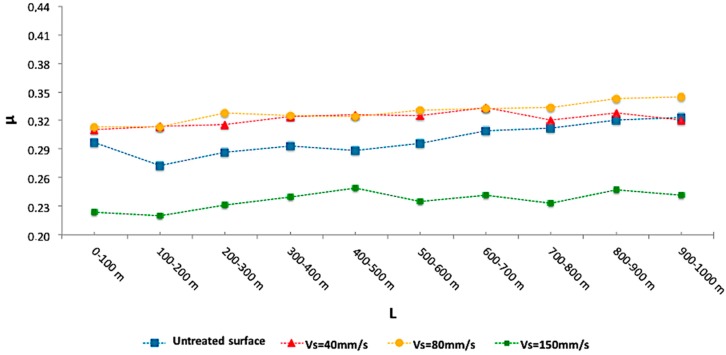
Friction coefficient for several surface treatments as a function of sliding distance.

Also, the analysis of friction coefficient (μ) evolution in the pin on disc tests, reveals the existence of a critical thickness of modified layer from which the advantages of the oxidative texture of the alloy are reduced. This fact, observed in preliminary studies, is mainly caused by the lack of compactness detected by SEM/EDS and the non-homogeneity of oxides by the treatment time and the cooling resulting, among others, in the appearance of surface cracks. In this case, treatments which produce the lowest friction coefficient were at scanning speeds of 150 mm/s, Figure 8. This decrease on friction coefficient is derived from a reduction of the surface finish values from attenuation of the initial asperities volume on the sliding path and its detachment, significantly conditioning the involved wear mechanisms.

By evaluating the relationship between the friction coefficient and the analysis of the sliding trace and pin after the tests, specific behaviors can be detected in the wear mechanisms. The identification and understanding of these mechanisms favors the selection of surface modification treatments focused on the reduction of wear and the increase of the tribological performance of materials.

By analyzing the friction coefficient on the first 250 m of sliding distance (L) from a pin on disc test, three different stages can be observed along the sliding path. These stages are associated with wear mechanisms that may explain the contact phenomena that takes place between the tribological pair elements.

In the first stage, a significant increase of the contact force and μ, habitual in this type of test, is caused due to the impact between the two types of materials. This rise of μ continues with a gradual decrease due to the relaxation of the materials, resulting in the recovery of the balance of forces after the first contact between pin and disc.

**Figure 9:**
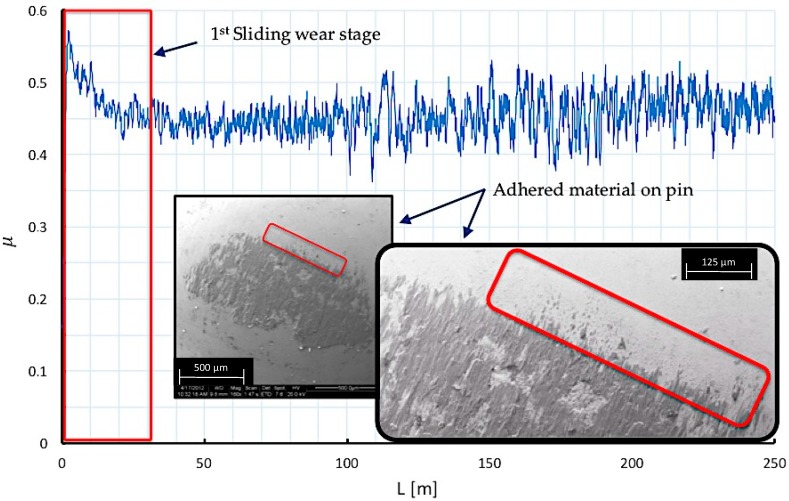
Wear mechanisms and friction coefficient behavior at the first stage of sliding distance.

From the point of view of the involved wear mechanisms, abrasion phenomena effects can be observed, due mainly to the detachment of initial asperities derived from the roughness induced by the textured treatment. These asperities, because of the smaller contact surface with the pin, give rise to higher contact pressures, tending to be eliminated when sliding occurs, Figure 9.

**Figure 10:**
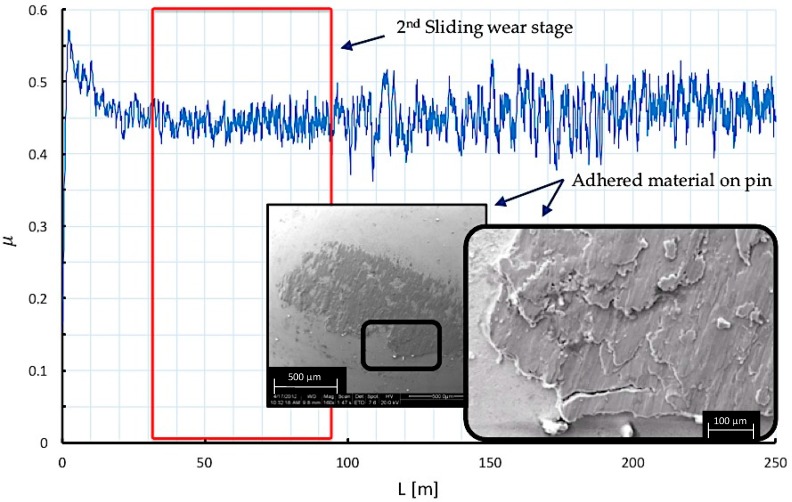
Wear mechanisms and friction coefficient behavior at the second stage of sliding distance.

The second stage of the test is associated with an increase in the volume of material removed in the disc, which favors the adhesion of material of the modified layer over the pin in stratified sections formed from the slip track. The contact force and friction coefficient describe variable oscillations due to the lack of homogeneity in the contact zone. Under these conditions, adhesion effects induce the modification of the surface topography of the contacting elements towards a softer topography, resulting in sliding with contact surfaces of the same material, and causing a decrease of the oscillations of μ, Figure 10. However, the increase of adhered material volume is produced until critical dimensions are reached, when the material cannot be supported, and the detachment of part of the adhered layer occurs. 

In the last stage of the test, from the observation of the evolution of forces involved in the process, an increase in the amplitude of the recorded friction coefficient values can be detected. The increase of amplitude implies a growth of the instability in the sliding trace due to the lack of uniformity of the circular groove.

In this phase, detachments of adhered material to the pin is produced as a result of reaching an excessive critical and unstable volume, which results in the incorporation of material debris into the sliding track of the outer layers of the disc (adhered in the first moments on the sphere), and WC-Co pin debris. This type of debris presents higher hardness values than untreated Ti6Al4V alloy, giving rise to abrasion phenomena on the wear groove. 

**Figure 11:**
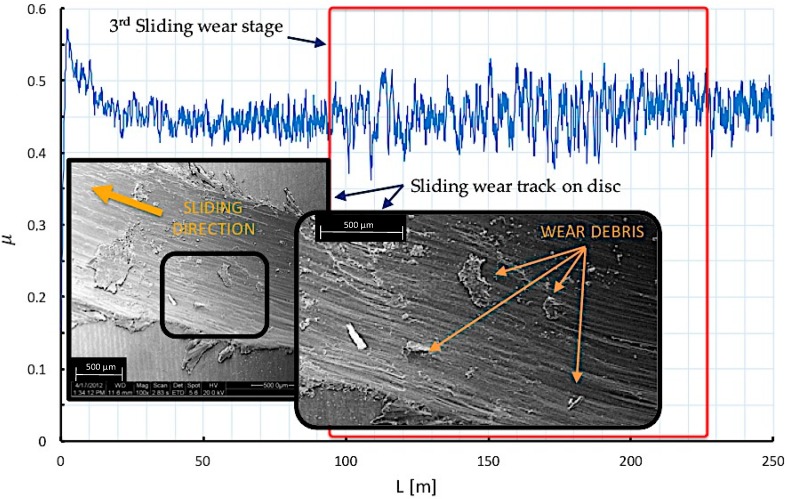
Wear mechanisms and friction coefficient behavior at the last stage of sliding distance.

Effects of three body abrasion and adhesion on the sliding trace are easily identifiable because the wear is logically is located following the circular trajectory of the pin displacement, Figure 11. As can be seen, it is a process that is repeated in which after the increasing amplitudes stage, a decrease of the amplitudes is produced, mainly due to the cycles of adhesion produced in the contact zone of the tribological pair.

Thus, after the analysis of results, a direct dependence of the alloy behavior under sliding wear conditions with respect to the laser treatment parameters can be confirmed, keeping the scan speed of the beam (Vs) as the process control variable. Using the friction coefficient as a reference, it is found that treatments performed at relatively high speeds (150 mm/s) result in lower friction coefficient values, reducing the effects of sliding wear of the alloy. Also, increases in Vs also affect the generation of softer topographies, with lower roughness values in terms of the Rpk parameter. However, it is remarkable that lower rates of Vs, with greater thickness of the treated layer, produce an increase in the concentration of oxides, modifying the initial color tonality of the alloy and increasing significantly the hardness of the material.

## 4. Conclusions

It has been shown that surface laser treatments on UNS R56400 (Ti6Al4V) develop Ti*_x_*O*_y_*, mainly TiO_2_, based compact coatings, which allow for the modification and improvement of surface properties such as coloration, roughness, and tribological features. Beam pulse frequency, F, and scanner speed, Vs, have been used as manufacturing parameters. The most advantageous results for the improvement of tribological behavior were detected in textured surfaces using a Vs of 150 mm/s, resulting in a decrease in the friction coefficient values by approximately 20%. Stereoscopic Optical Microscopy (SOM) and SEM analysis demonstrated that roughness (measured through Rpk) of the modified layer can be related with Vs. Thus, higher Vs values induce lower asperities height and reduce Rpk because the beam is applied for a limited time on the surface. This fact favors the design and development of topographies for obtaining high sliding-friction-wear resistance. Therefore, 150 mm/s shows a remarkable decrease in the friction coefficient of the treated layer, promoting wear improvements to the alloy under friction and sliding conditions.

Introduction of oxides on the alloy surface by means of texturing, where the cooling process takes place in a short period of time, results in an increase of hardness, maintaining also a direct dependence with Vs. In this way, an area close to the textured trace is observed on cross section of the samples that shows an increase in the concentration of oxides and produces an important increase of the hardness values.

## Figures and Tables

**Table 1:** UNS R56400 alloy weight composition (wt %) obtained by Optical Emission Spectrometry (OES).

Al	V	Fe	C	O	N	H	Ti
6.26	3.91	0.18	0.011	<0.10	<0.10	<0.10	Rest

**Table 2:** Laser texturing parameters. P = Power; F = pulse rate; Vs = scan speed of the beam.

P (W)	F (kHz)	Vs (mm/s)							
10	20	10	20	40	80	100	150	200	250
	50								
8	70	40			80			150	
